# Prediction of phonon-mediated superconductivity in new Ti-based M$_2$AX phases

**DOI:** 10.1038/s41598-022-17539-8

**Published:** 2022-08-01

**Authors:** E. Karaca, P. J. P. Byrne, P. J. Hasnip, M. I. J. Probert

**Affiliations:** 1 Department of Physics, University of York, York, YO10 5DD UK; 2 Biomedical, Magnetic and Semiconductor Materials Research Center (BIMAS-RC), Sakarya University, 54187 Sakarya, Turkey

**Keywords:** Superconducting properties and materials, Density functional theory, Electronic structure

## Abstract

A high-throughput computational method is used to predict 39 new superconductors in the Ti-based M$_2$AX phases, and the best candidates are then studied in more detail using density functional theory electron–phonon coupling calculations. The detailed calculations agree with the simple predictions, and Ti$_2$AlX (X: B, C and N) materials are predicted to have higher values of $T_c$ than any currently known hexagonal M$_2$AX phases. The electronic states at the Fermi level are dominated by the Ti 3d states. The choice of X (X: B, C and N) has a significant impact on the electronic density of states but not on the phonon characteristics. The electron–phonon coupling parameter for Ti$_2$AlX (X: B, C and N) was determined to be 0.685, 0.743 and 0.775 with a predicted $T_c$ of 7.8 K, 10.8 K and 13.0 K, respectively.

## Introduction

MAX phases are hexagonal carbides or nitrides with the chemical formula M$_{n+1}$AX$_n$^1^ where n = 1, 2, 3, etc. Here, M is an early transition metal, A is mainly group 13–16 and X is either C or N. MAX phases exhibit high damage tolerance, excellent thermal shock resistance, resistance to corrosion and oxidation, high creep lifetime, exceptionally damage-tolerant good machinability, and are electrically and thermally conductive^2–12^. The physical properties of hexagonal carbides and nitrides in the ternary M$_2$AX family phase have been extensively studied due to their unusual combination of properties typically associated with metals and ceramics. Additionally, in the last 2 years, some M$_2$AX phase ceramics, including B as the X element have been synthesized for the first time^13–16^. Boron and its compounds have important technological uses due to their interesting physical and chemical properties^17,18^. As such, MAX phase borides are also expected to become promising research and application candidates, particularly in the nuclear industry due to their enhanced stability^19^. Hadi et al. investigated the impact of substituting B for C and N in Nb$_2$SX(X:B, C and N) on the structural, electronic, mechanical, thermal and optical properties. They discovered that compared to Nb$_2$SC and Nb$_2$SN, Nb$_2$SB was mechanically stronger, more covalent, more resistant to shear deformation and more elastically and optically isotropic^19^. Some M$_2$AX phases are superconductors, with the highest known $T_c$ in Nb$_2$GeC which has $T_c$ = 10 K^20^.

The purpose of this study is to predict new superconducting materials within the Ti-based M$_2$AX family and increase the maximum $T_c$. In addition, we present the first study of superconductivity in the recently synthesized boride-based M$_2$AX phases. This is a proof of principle of our high-throughput method to quickly screen M$_2$AX superconductors to provide useful guidance for experiments.

Three materials (Ti$_2$GeC, Ti$_2$InC and Ti$_2$InN) were initially studied and compared to known experimental $T_c$ data to create a high-throughput screening model for Ti-based materials, based on the Fröhlich model we developed to predict $T_c$ in Nb-C based M$_2$AX phases^21^. We then used this model to screen 42 different Ti$_2$AX materials (where A: Al, Si, P, S, Cu, Zn, Ga, Ge, As, Cd, In, Sn, Tl and Pb; X: B, C and N). For each X it was found that A = Al gave the highest predicted $T_c$.

In 1963, Jeitschko et al. reported on the manufacture and characterisation of Ti$_2$AlN^22^, from which the hexagonal M$_2$AX phase family developed. So far, about 60 M$_2$AX phases have been synthesized^1^, but only 10 of them have been shown to be superconductors in experiment: Mo$_2$GaC (4.0 K)^23^, Nb$_2$SC (5.0 K)^24^, Nb$_2$AsC (2.0 K)^25^, Nb$_2$SnC (7.8 K)^26^, Ti$_2$InC (3.1 K)^27^, Nb$_2$InC (7.5 K)^28^, Ti$_2$InN (7.3 K)^29^, Ti$_2$GeC (9.5 K)^30^, Lu$_2$SnC (5.2 K)^31^ and Nb$_2$GeC (10.0 K)^20^. Of these, Nb$_2$GeC has the highest known $T_c$=10 K. Attempts to synthesize V$_2$AlN have only succeeded in growing the related cubic non-MAX phase, which has recently been shown to be superconducting with $T_c$ = 15.9 K^32^. The M$_2$AX phases have very useful mechanical properties, including high damage tolerance, excellent thermal shock resistance, resistance to corrosion and oxidation, high creep lifetime, and good machinability, which are properties that are not often found in superconductors, and so could have novel applications.

As our high-throughput model predicted the highest $T_c$ for Ti$_2$AlX (X: B, C and N), we therefore performed a more detailed study of superconductivity in these materials Whilst the structural, electronic, elastic, thermodynamic, and vibrational properties of Ti$_2$AlX (X: B, C and N) have been studied theoretically^33–37^ and experimentally^35,38^, we are unaware of any study of superconductivity in these materials. Thus, this work presents an ab initio study of the superconducting $T_c$, including electron-phonon coupling, as well as structural, electronic and phonon properties of Ti$_2$AlX (X: B, C, and N), using the plane-wave pseudopotential approach to density functional theory (DFT). The Eliashberg spectral function is calculated by combining linear response theory^39,40^ with Migdal–Eliashberg theory^41,42^. These quantities are then used to investigate the origin of superconductivity in these materials, and the effect of changing X (X: B, C, and N). In our previous study of superconductivity in Nb-C based M$_2$AX phases^21^ we found that the Migdal–Eliashberg predictions agreed with experimental $T_c$ values within $\pm\, 1$ K.

## Methods

The calculations used the Quantum Espresso ab initio simulation package^39,40,43^ with the Perdew–Burke–Ernzerhof (PBE)^44^ exchange-correlation approximation and ultrasoft pseudopotentials^45^. The plane-wave basis cut-off is 60 Ry ($\sim$ 812 eV) and the Brillouin zone integration used the Monkorst-Pack^46^ scheme with ($36 \times 36 \times 8$) **k**-mesh (maximum spacing of 0.01 $\times 2 \pi \text{\AA} ^{-1}$) whilst electronic and Fermi surface calculations are performed with a denser ($40 \times 40 \times 10$) **k**-mesh.

Phonon calculations used the linear response approach^39,40,43^ and the Brillouin zone integration for the phonons used a ($4 \times 4 \times 4$) **q**-mesh and twelve dynamical matrices by symmetry. The electron and phonon results are combined to compute the electron-phonon interaction using the Migdal-Eliashberg theory^41,42^ and hence $T_c$.

This calculation of $T_c$ is very computationally demanding, and is therefore impractical for high-throughput screening for novel superconductors. A change in the A element in Ti$_2$AX (X: B, C, and N) appears to have a comparable impact to the superconducting isotope effect, and in our previous work^21^ on Nb-C based M$_2$AX phases, we showed that a simple model based upon the Fröhlich^47^ theory of the isotope effect was an effective foundation for a high-throughput screening approach of these materials, with

1 $$\begin{aligned} T_c = \alpha \frac{N(E_{F})}{\sqrt{M}} - T_0, \end{aligned}$$

where M is the mass of a formula unit, N(E$_F$) is the electronic density at the Fermi energy E$_F$ and T$_0$ and $\alpha$ are linear fit parameters. This model has a critical value of $N(E_F)/\sqrt{M} > T_0/\alpha$ for superconductivity to occur. This functional form is an approximation to the simplified BCS equation^48^.

In its most basic form, BCS theory gives the superconducting transition temperature $T_c$ in terms of the electron–phonon interaction (*V*) and the Debye temperature ($\Theta _D$), and can be simplified as

2 $$\begin{aligned} T_c = 1.134 \Theta _D \exp \left( \frac{-1}{N(E_{F})V}\right) , \end{aligned}$$

where $\Theta _D\sim 1/\sqrt{M}$. This exponential form is approximately linear when $0.2< NV < 0.7$ and saturates at large values of *NV*. Within each Ti$_2$AX family (borides, carbides and nitrides) we might expect a similar *V* and hence observe behaviour similar to Eq. (1).

The advantage of Eq. (1) is that N(E$_F$) can be calculated in much less time (typically less than 1 core hour) than the electron–phonon matrix elements (typically 300 core hours per material), and when combined with the observed trend in $T_c$ *vs.* $N(E_F)/\sqrt{M}$ for known superconducting materials (Ti$_2$GeC, Ti$_2$InC and Ti$_2$InN), it can be used in a high-throughput search to predict the superconducting transition temperatures of candidate materials for which there have been no previous superconductivity studies. The most promising of these is Ti$_2$AlX(X: B, C and N) which is then investigated in more detail using full electron–phonon coupling and Migdal–Eliashberg theory.

## Results

### Superconducting $T_c$ results

**Figure 1:**
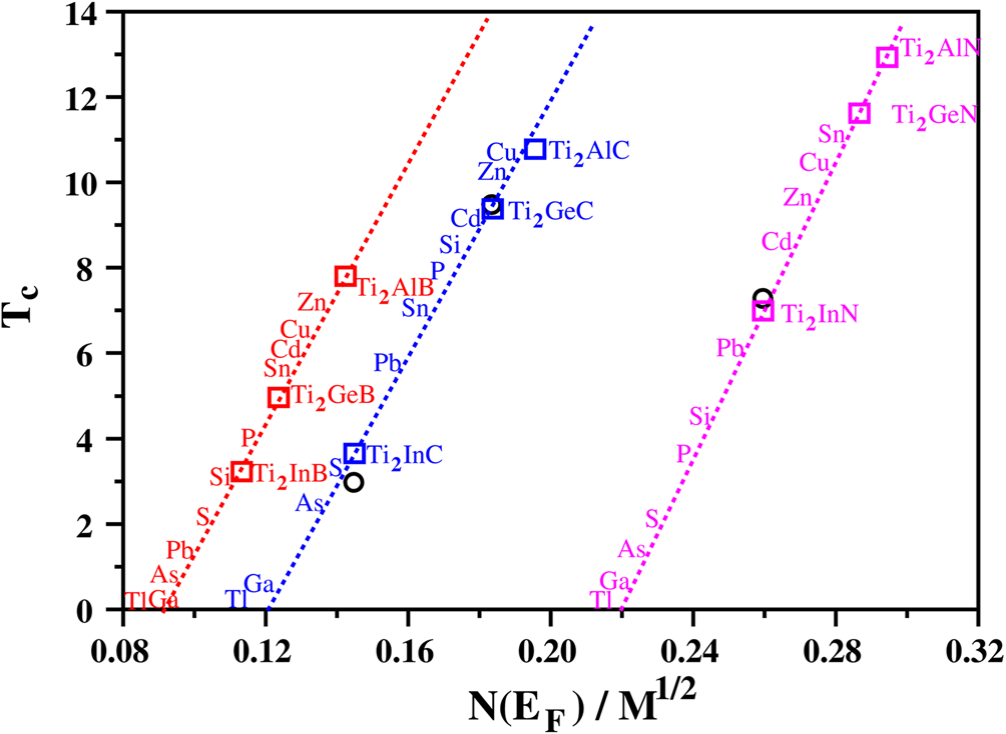
For Ti$_2$AX (A: Al, Ge and In; X: B, C and N), the results for $T_c$ computed using $\mu ^*=$ 0.13 using Migdal–Eliashberg theory are presented as red (X = B), blue (X = C) and magenta (X = N) squares, and the corresponding experimental data are displayed as black circles, with linear best fit to the theoretical values in red, blue and magenta dashed lines. Red, blue and magenta dashed lines represent a simple Fröhlich model for estimating superconducting transition temperature $T_c$. Full data in Tables 1 and 2.

The high-throughput screening is based on our Fröhlich model, which predicts a linear relationship between the critical temperature ($T_c$) and the value of N(E$_F$)/$\sqrt{M}$ (as shown in Fig. 1). As shown in the Supplementary Information, the $T_c$ values of three known superconductors (Ti$_2$GeC, Ti$_2$InC and Ti$_2$InN) were calculated using the Eliashberg theory^41,42^ and the best fit to experimental $T_c$ values is found when $\mu ^{*}=$ 0.13 for all 3 materials. These theoretical values are shown in Fig. 1 using blue and magenta squares, whilst the corresponding experimental $T_c$ values are given in black circles.

In our previous work^21^ we found that Nb-C based M$_2$AX phases containing Al had high $T_c$ values. Hence we used the Eliashberg theory with $\mu ^{*}=$ 0.13 to determine the superconductivity temperatures of Ti$_2$AlX(X: C and N) and Ti$_2$GeN materials, none of which have a known $T_c$. These results are also plotted in Fig. 1 as blue and magenta squares, and it appears that the M$_2$AX carbides and nitrides fall into 2 distinct classes, which we have shown by 2 straight lines.

**Table 1:** Superconducting temperature values ($T_c$ in K) of experimental, predicted through simple Fröhlich model and calculated by Migdal–Eliashberg (ME) theory for Ti$_2$AX (A: Al, Ge and In, X: B, C and N).

Phase	$T_{c}$ (Exp)	$T_{c}$ ( Fröhlich)	$T_{c}$ (Full ME theory)
Ti$_2$AlB		7.8	7.8
Ti$_2$GeB		4.9	5.0
Ti$_2$InB		3.4	3.2
Ti$_2$AlC		11.2	10.8
Ti$_2$GeC	9.5^30^	9.3	9.4
Ti$_2$InC	3.1^27^	3.6	3.7
Ti$_2$AlN		12.9	13.0
Ti$_2$GeN		11.6	11.6
Ti$_2$InN	7.3^29^	6.9	7.0

Recently, a new family of boride M$_2$AX phases^49^ has been synthesized but has yet to be tested for superconductivity. We therefore used Eliashberg theory to calculate the superconductivity temperatures of Ti$_2$AB(A: Al, Ge, In) using $\mu ^{*}=$ 0.13 as before, for a direct comparison with the carbides and nitrides. These results are also plotted in Fig. 1 as red squares, and it appears that the borides fall into a third distinct class.

With electron–phonon coupling calculation results of $T_c$ for 3 materials in each of the Ti-based boride/carbide/nitride families, we can perform a simple linear fit and use this as the basis for our Fröhlich model. The results are summarized in Table 1, and validate the usefulness of our screening method to predict $T_c$ in novel materials.

We can now use this model in a high-throughput screening approach to quickly evaluate similar materials for which $T_c$ is unknown. This only requires an electronic DOS calculation ($\sim$ 1 core hour/material) which is much quicker than calculating the full electron–phonon coupling ($\sim$ 300 core hours/material).

The full set of Fröhlich model results, for Ti$_2$AX (where A: Al, Si, P, S, Cu, Zn, Ga, Ge, As, Cd, In, Sn, Tl and Pb; X: B, C and N) are shown in Table 2. This shows that the compounds containing Al have the highest superconducting transition temperature for each family, and that Ti$_2$AlN is predicted to have the highest superconductivity temperature of *any* known M$_2$AX material. Similarly, Ti$_2$AlC is predicted to have the highest $T_c$ of the carbides, and Ti$_2$AlB is predicted to have the highest $T_c$ of the borides.

**Table 2:** Superconducting temperature values of all the different M$_2$AX phases screened by the high-throughput Fröhlich model.

Phase	$N(E_F)$ (States/eV)	$\sqrt{M}$ (amu$^{0.5}$)	$T_{c}$ (K)
Ti$_2$AlB	2.241	16.3	7.8
Ti$_2$SiB	1.758	16.4	2.9
Ti$_2$PB	1.902	16.5	3.9
Ti$_2$SB	1.703	16.6	2.1
Ti$_2$CuB	2.354	18.4	6.4
Ti$_2$ZnB	2.446	18.5	7.1
Ti$_2$GaB	1.718	18.8	0.3
Ti$_2$GeB	2.227	18.9	4.9
Ti$_2$AsB	1.799	19.1	0.6
Ti$_2$CdB	2.631	20.9	6.1
Ti$_2$InB	2.324	21.0	3.4
Ti$_2$SnB	2.617	21.2	5.7
Ti$_2$TlB	2.066	24.9	0.0
Ti$_2$PbB	2.440	25.0	1.2
Ti$_2$AlC	3.048	16.4	11.2
Ti$_2$SiC	2.788	16.5	8.4
Ti$_2$PC	2.727	16.6	7.6
Ti$_2$SC	2.314	16.7	3.0
Ti$_2$CuC	3.335	18.5	10.5
Ti$_2$ZnC	3.328	18.6	10.1
Ti$_2$GaC	2.226	18.8	0.3
Ti$_2$GeC	3.319	19.0	9.3
Ti$_2$AsC	2.506	19.1	2.4
Ti$_2$CdC	3.599	21.0	9.1
Ti$_2$InC	2.938	21.1	3.6
Ti$_2$SnC	3.427	21.3	7.1
Ti$_2$TlC	2.730	25.0	0.0
Ti$_2$PbC	3.791	25.1	5.7
Ti$_2$AlN	4.568	16.5	12.9
Ti$_2$SiN	3.864	16.6	4.3
Ti$_2$PN	3.796	16.7	3.6
Ti$_2$SN	3.688	16.8	2.0
Ti$_2$CuN	4.890	18.6	10.3
Ti$_2$ZnN	4.841	18.7	9.7
Ti$_2$GaN	3.991	18.9	0.4
Ti$_2$GeN	5.144	19.1	11.6
Ti$_2$AsN	4.110	19.2	1.4
Ti$_2$CdN	5.321	21.1	8.4
Ti$_2$InN	5.182	21.2	6.9
Ti$_2$SnN	5.672	21.4	11.0
Ti$_2$TlN	5.084	25.1	0.0
Ti$_2$PbN	6.043	25.2	6.0

**Figure 2:**
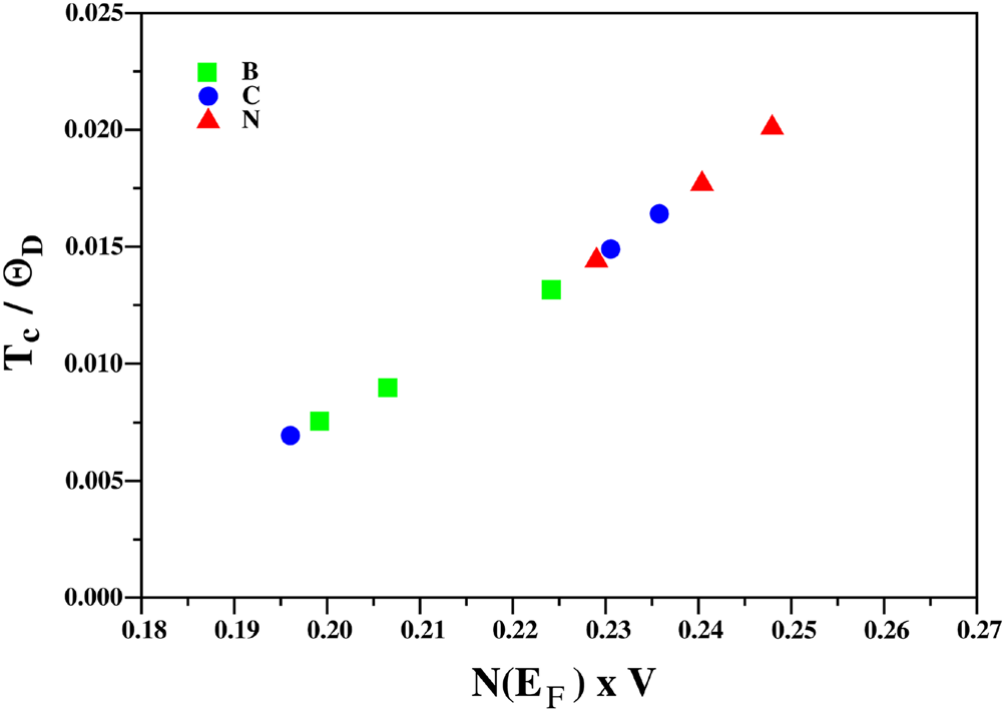
Simple BCS theory analysis of the observed trends in $T_c$ for Ti$_2$AX (A: Al, Ge, In, and X: B, C, or N).

As a validation of Eq. (1), we calculated values for $\Theta _D$ using Quantum Espresso’s QHA package, and these are shown in Table 3 along with the values for $T_c$ and $N(E_{F})$. This allows the electron phonon interaction strength (*V*) to be inferred from Eq. (2). This shows that the borides/carbides/nitrides all follow the same universal form as shown in Fig. 2.

**Table 3:** The effective electron–phonon coupling potential $N(E_F)V$ and calculated values for the Debye temperature, $\Theta _D$, for the MAX phases studied.

Phase	$N(E_{F}) V$	$\Theta _D$ (K)
Ti$_2$AlB	0.224	594.3
Ti$_2$GeB	0.207	555.6
Ti$_2$InB	0.199	429.3
Ti$_2$AlC	0.231	722.2
Ti$_2$GeC	0.236	572.9
Ti$_2$InC	0.196	527.7
Ti$_2$AlN	0.240	728.9
Ti$_2$GeN	0.248	576.9
Ti$_2$InN	0.229	484.8

Having presented the $T_c$ results, we now consider the detailed electronic and phonon properties that underly this behaviour.

### Structural, electronic and Fermi surface properties

**Figure 3:**
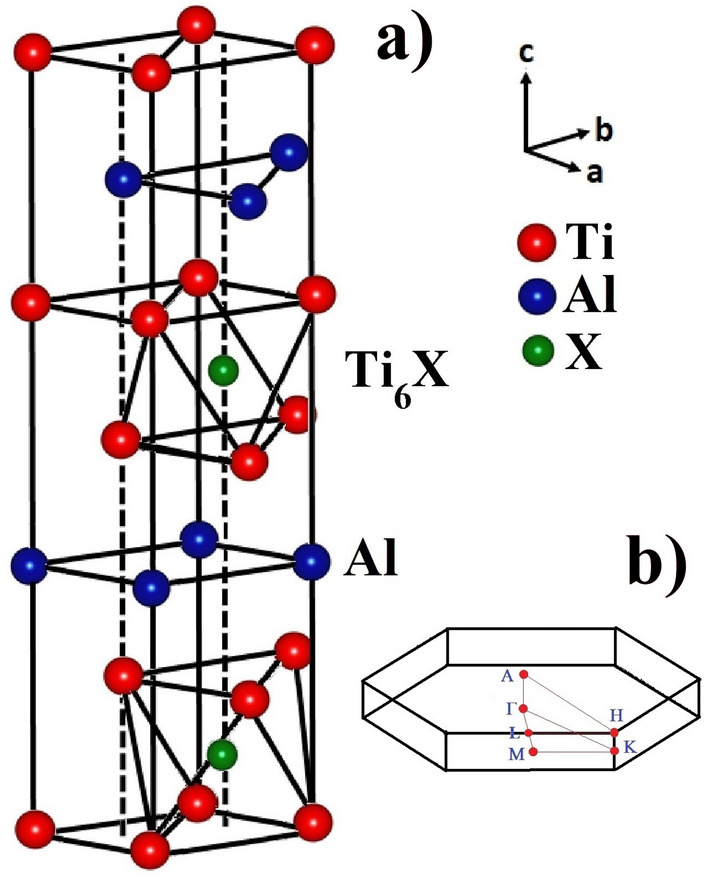
(**a**) The hexagonal crystal structure of Ti$_2$AlX (X: B, C and N) , where blocks of Ti-X (X: B, C and N) (formed by edge-shared Ti$_6$X (X: B, C and N) octahedra) are sandwiched with Al atomic sheets. (**b**) The hexagonal Brillouin zone for Ti$_2$AlX (X: B, C and N).

Ti$_2$AlX (X: B, C and N) crystallizes in the hexagonal structure with space group P6$_3$/mmc. The primitive unit cell has two formula units (eight atoms), each of which has occupied Wyckoff coordinates 4f (1/3, 2/3, *z*) for Ti, 2d (1/3, 2/3, 3/4) for Al, and 2a (0, 0, 0) for B (C and N) atoms. Thus, two lattice parameters, *a* and *c*, and one internal structural parameter, *z*, determine the structure. The hexagonal unit cell is shown in Fig. 3a in which blocks of edge-sharing Ti$_6$X octahedra are sandwiched between planes of Al. The hexagonal Brillouin zone is shown in Fig. 3b.

The Murnaghan equation of state was used to compute the lattice constants (*a*,*c*), the optimum internal parameter (*z*) and the bulk modulus (*B*) for each compound. These are shown in Table 4 and are in excellent agreement with previous theoretical results^33,34,36,37^.

**Table 4:** Structural properties of Ti$_2$AlX (X: B, C and N) for this work in bold, and their comparison with previous theoretical results.

Source	*a*(Å)	*c*(Å)	*z*	B(GPa)
**Ti**$_2$**AlB**	**3.153**	**14.359**	**0.086**	**110.7**
GGA^36^	3.148	14.077		111.9
GGA^37^	3.148	14.064	0.087	134.0
**Ti**$_2$**AlC**	**3.069**	**13.643**	**0.083**	**137.6**
Theory^33^	3.071	13.726		138.0
Theory^34^	3.040	13.600		
Theory^35^	3.040	13.600	0.084	
GGA^36^	3.069	13.737		140.3
**Ti**$_2$**AlN**	**2.995**	**13.722**	**0.085**	**155.7**
Theory^33^	2.998	13.634		155.0
Theory^34^	2.989	13.614		
GGA^36^	2.996	13.643		160.2

**Figure 4:**
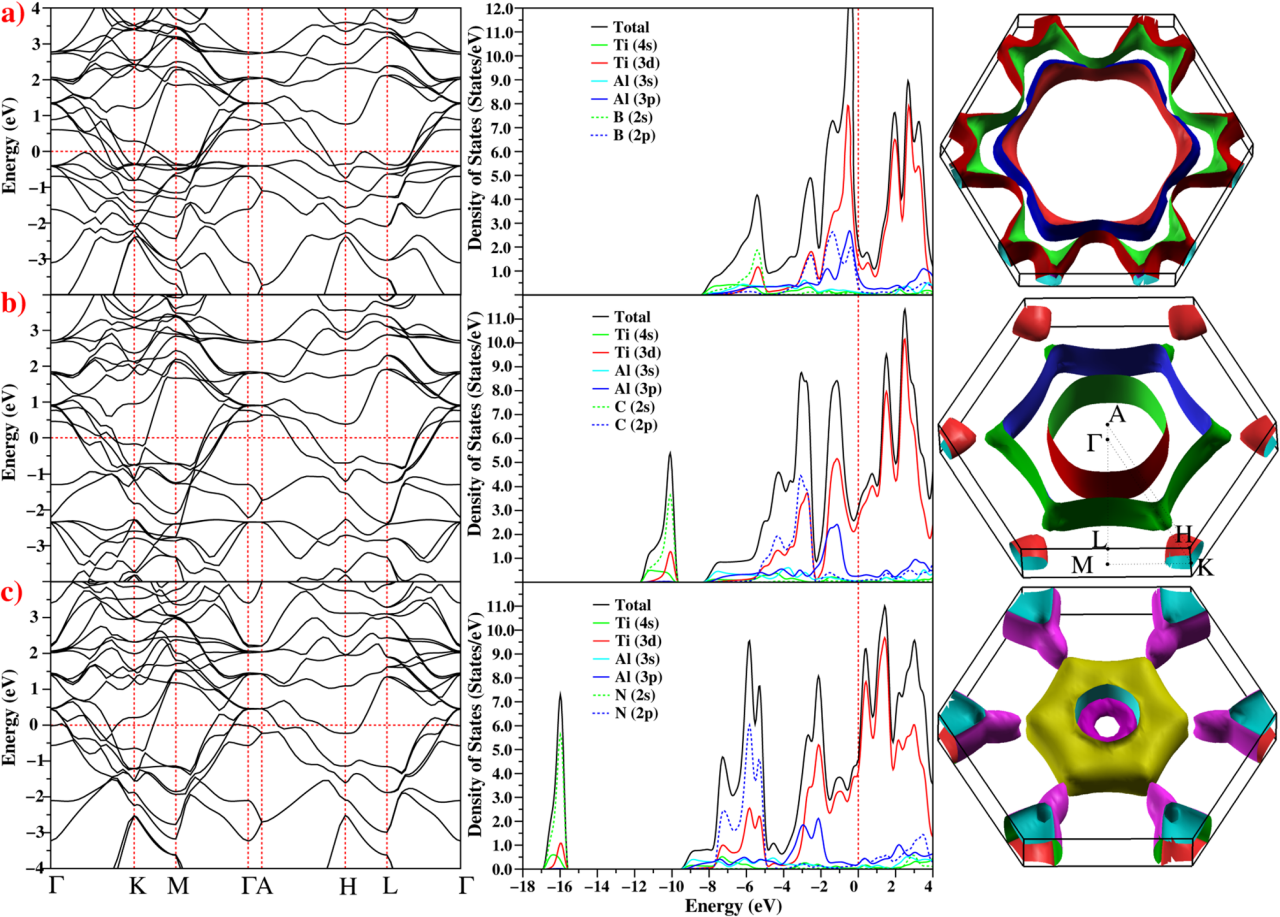
The electronic band structure, the total and partial electronic local density of states and Fermi surface for the hexagonal phase of (**a**) Ti$_2$AlB, (**b**) Ti$_2$AlC and (**c**) Ti$_2$AlN.

Figure 4 shows the electronic properties of hexagonal Ti$_2$AlX including the band structure in the Brillouin zone, the total and partial density of states (DOS and PDOS), and the Fermi surface. The electronic density of states at the Fermi level (N(E$_F$)) is important for metallic phases and superconductivity calculations. The PDOS of each component, broken into site and angular momentum contributions, is shown in Fig. 4, and as bands (mostly Ti 3d states) cross the Fermi level, this is the origin of the metallic behaviour. The electrical band structure is similar to that observed in previous studies^35,37^. Notably, each material has six valence bands that cross the Fermi level.

An analysis of the PDOS of Ti$_2$AlN reveals that the lowest energy region, ${-\,16.8< E < -\, 15.6}$ eV, is dominated by the N 2s states with minor contributions from Ti 4s and 3d states. In Ti$_2$AlC this low-energy region is dominated by the C 2s orbital and is about 5 eV higher than in Ti$_2$AlN. This low-energy region is absent in Ti$_2$AlB material.

The main valence band region, $-\, 8.0< E < -\, 4.0$ eV, is made of hybridised Ti 3d and X 2p states, indicating covalent Ti-X bonding, with increasing covalent character in the order of $B>C>N$. Hence it is expected that Ti$_2$AlN should have higher electrical conductivity than Ti$_2$AlB and Ti$_2$AlC. The region $-\, 4.0< E < E_F$ eV is dominated by Ti 3d states with some weak Ti 3d and Al 3p hybridization. In the region $E>E_F$ the band structure is almost entirely dominated by Ti 3d states.

Qualitatively, it appears that the band structures around the Fermi level for Ti$_2$AlX (X = B, C or N) are similar, with the principal difference being a shift of E$_F$ by 0.5 eV from X = B to X = C, and by 0.2 eV from X = C to X = N. This shift results in a significant increase in N(E$_F$). The electronic states at the Fermi level are crucial for superconductivity and we find that the DOS of Ti$_2$AlN at the Fermi level is N(E$_F$) = 4.568 states/eV, with about 89.3%, 5.9% and 4.8% contributions from Ti, Al, and N atoms, respectively. As a consequence, the conduction properties are dominated by the Ti 3d electrons. Similarly, N(E$_F$)= 3.048 states/eV for Ti$_2$AlC and 2.241 states/eV for Ti$_2$AlB. This suggests that the most important contribution to the formation of the superconducting properties of Ti$_2$AlX phases come from the Ti 3d states which increase N(E$_F$) and enhance $\lambda$ according to the McMillan–Hopfield expression^50^:

3 $$\begin{aligned} \lambda =\frac{N(E_{F})\langle I^2 \rangle }{M\langle \omega ^2 \rangle }, \end{aligned}$$

where $\langle \omega ^2 \rangle$ denotes the average squared phonon frequency, $\langle I^2 \rangle$ describe the average squared electron–phonon matrix element and M is the average atomic mass. As Ti$_2$AlN has higher N(E$_F$) than the other materials, it should result in a higher $T_c$ value if all other effects are similar.

Figure 4 also shows the Fermi surface of Ti$_2$AlX (X: B, C and N) which again illustrates the dominance of Ti 3d-like bands. The calculated Fermi surface of Ti$_2$AlC agrees well with the previous theoretical result^35^. The Fermi surface of Ti$_2$AlB has four sheets, while the Fermi surface of the other two materials contains five sheets. The Fermi surface is completely prismatic and cylindrical in the $\Gamma$-*A* direction and exhibits electron-like behaviour, while hole-like sheets appear at the corners of the Brillouin zone along the *H*-*K* and *L*-*M* directions. The non-spherical Fermi sheets may also cause a high metallic conductivity^51^.

### Phonons and electron–phonon interaction

**Figure 5:**
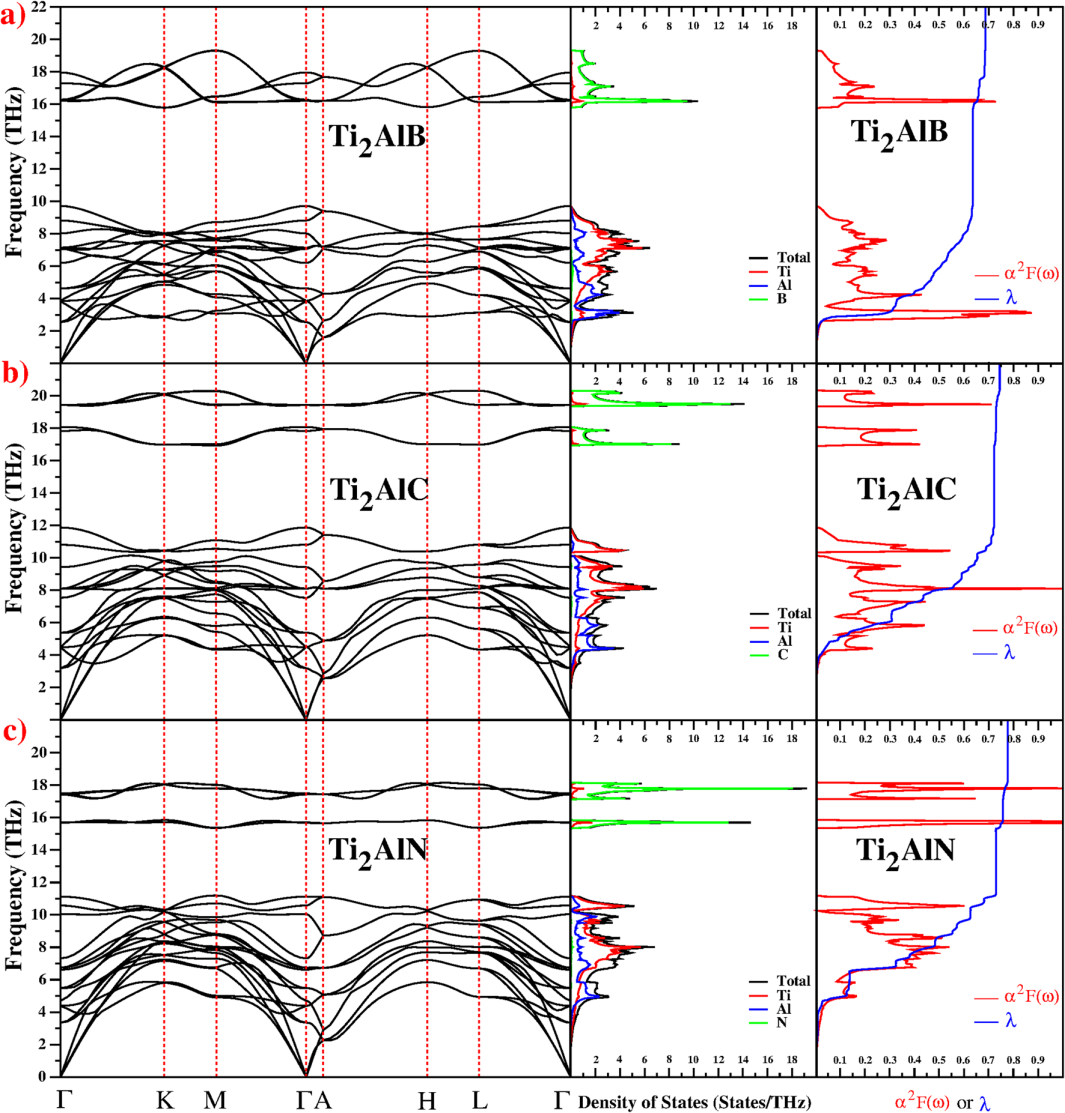
Phonon dispersion curves, total, partial vibrational density of states and the calculated electron-phonon spectral function $\alpha ^2 F(\omega )$ (red line) and the variation of the electron–phonon coupling parameter (blue line) with rising frequency $\lambda$($\omega$) of (**a**) Ti$_2$AlB, (**b**) Ti$_2$AlC and (**c**) Ti$_2$AlN.

Phonons have a crucial role in superconductivity, so the calculated phonon dispersion, total and partial vibrational density of states and electron–phonon spectral function for Ti$_2$AlX (X: B, C and N) are shown in Fig. 5a–c, respectively. All 3 materials have 8 atoms per primitive unit cell, so there are 3 acoustic and 21 optical phonon modes. A detailed study of the zone centre optical phonon modes is given in our previous paper^21^. As there are no negative frequencies, each structure is dynamically stable. The phonon spectra splits into two distinct frequency regions: a low-frequency region up to 12 THz that contains three acoustic and fifteen optical phonon modes, and a high-frequency region from 15 to 21 THz that contains six optical modes. The $E_{2g}$ branch of all three materials exhibits a phonon anomaly along the $\Gamma$-*K* direction.

There is weak overlap and hybridization of Ti–Al modes in the low-frequency region, and the modes in the high frequency region are dominated by the light X atoms. The DOS in this region has two peaks separated by a small gap for the atoms of Ti$_2$AlC and Ti$_2$AlN compounds but this gap vanishes for Ti$_2$AlB. Overall, the phonon properties of Ti$_2$AlX (X: B, C and N) materials are very similar.

The electron–phonon interaction may be studied using the linear response theory^39,40^ approach to the Migdal–Eliashberg theory^41,42^. The average electron-phonon coupling constant $\lambda$ may be calculated from the Eliashberg spectral function ($\alpha ^2F(\omega )$). A detailed study of the Eliashberg spectral function is given in our previous paper^21^. $\lambda$ values of Ti$_2$AlX (X: B, C and N) have been calculated as 0.685, 0.743 and 0.775, respectively. Figure 5 confirms that $\lambda (\omega )$ is dominated by the lowest frequency region, in which $\lambda \propto \omega$. The low frequency contribution to the total $\lambda =$ is 93%, 97% and 94%, respectively, and is dominated by the coupled motion of Ti and Al atoms. The high-frequency region makes a minor contribution to $\lambda$ as this region is dominated by light X atom modes. Using the value of $\lambda (\omega )$, the logarithmic average phonon frequency ($\omega _{\ln }$) is calculated as 219.540 K, 342.218 K and 369.818 K for Ti$_2$AlX (X: B, C and N), respectively. The values of $\lambda$ and $\omega _{\ln }$ are used to calculate the superconducting transition temperature $T_c$ using the Allen–Dynes modification of the McMillan formula as discussed in our previous paper^21^. In most studies, the value of $\mu ^{*}$ ranges from 0.10 to 0.16^50,52^. Here, we use $\mu ^{*}$=0.13, as this gave the best fit to the experimental $T_c$ for Ti$_2$GeC, Ti$_2$InC, and Ti$_2$InN (see Supplementary Information). There is currently no known experimental $T_c$ for Ti$_2$AlX and so we use $\mu ^{*}=$ 0.13 and predict $T_c =$ 7.8, 10.8, and 13.0 K for Ti$_2$AlX (B, C, and N).

## Conclusion

We have used a high-throughput approach to study the superconducting properties of 42 different Ti-based M$_2$AX phases where A: Al, Si, P, S, Cu, Zn, Ga, Ge, As, Cd, In, Sn, Tl and Pb; X: B, C and N; as shown in Fig. 1 and detailed in Table 2. Currently, 3 are known to be superconducting in experiment (black circles in Fig. 1). Our screening identified that A = Al has the best potential for a high $T_c$, and we then studied the properties of Ti$_2$AlX (X:B, C and N) in more detail. The M$_2$AX phase with the highest known experimental $T_c$ is Nb$_2$GeC which has $T_c = 9.5~\text {K}$. Our study predicts that Ti$_2$AlC will have $T_c = 10.8~\text {K}$, the highest $T_c$ for a carbide-based M$_2$AX phase. Our high-throughput model also predicts the potential for even higher $T_c$ in the nitride-based materials, and our detailed calculations predict Ti$_2$AlN to have $T_c = 13.0~\text {K}$. We also demonstrate superconductivity in the boride-based M$_2$AX phases for the first time.

Our analysis shows that the electron-phonon coupling is dominated by low-frequency Ti-based phonon modes, and Ti 3d-based electronic states near the Fermi energy. This work should encourage further studies of superconductivity in M$_2$AX phases, and the use of Al rather than the more usual Ge or In should have higher $T_c$ and cost savings.

The high-throughput model developed here, with its detailed justification, should also have application in other systematic studies of superconductivity.

## Supplementary Information

Supplementary Information.

## Data Availability

The data created and analysed during the current study are available from the corresponding author on reasonable request.
