# Supplementary material for: Prediction of phonon-mediated superconductivity in new Ti-based M2AX phases
Source: Sci Rep. 2022 Aug 1;12:13198. doi: 10.1038/s41598-022-17539-8 (PMC9343435; doi:10.1038/s41598-022-17539-8)
Supplement: Supplementary file 1 — Supplementary Information. [file 41598_2022_17539_MOESM1_ESM.pdf]

Supplementary Material: Prediction of Phonon-Mediated Superconductivity in New Ti-based M_2AX Phases

Karaca, E.[1,2]; Byrne, P. J. P.[1]; Hasnip, P.J.[1] and Probert, M.I.J.[1]
[1] *Department of Physics, University of York,*
York YO10 5DD, United Kingdom
and
[2] *Sakarya University, Biomedical,*
Magnetic and Semiconductor Materials Research Center (BIMAS-RC), 54187, Sakarya, Turkey

A. Structural, electronic and Fermi surface properties of Ti_2AX (A: Ge, In and X: B, C or N) compounds

We investigated the structural, electrical, Fermi surface, phonon and electron-phonon properties of Ti_2GeC, Ti_2InC, and Ti_2InN materials with previously known experimental superconducting transition temperatures T_c. The hexagonal structure of Ti_2AX (A: Ge, In and X: B, C or N) crystallizes in the space group $P6_3/mmc$. The main text discusses the crystal structure properties of Ti_2AlX (X: B, C, or N), which is same as Ti_2AX (A: Ge, In and X: B, C or N). The calculated equilibrium lattice constants (a,c), internal parameter (z), Ti–Ti, Ti–A(A:Ge and In), Ti–X (X: B, C and N) distances, bulk modulus (B) and its pressure derivative (B') have been determined and compared to existing experimental and theoretical data in table S1. These results are in good agreement with previous known experimental and theoretical results[1–7] although there has been no publications on Ti_2GeB material to date. All the Ti-Ti distances are longer than the elemental Ti distance (2.80 Å). Ionic and covalent characteristics are apparent in the bond lengths between Ti-X (X: B, C, and N) elements in each material, as the total of their covalent radii is bigger than the bond lengths between Ti-X atoms in these phases. The shorter atomic distance between Ti and X (where B, C, and N are present) implies the covalent bonding between Ti and X is stronger than Ti-Al. As a result, the Ti-X (X = B, C and N) bond is stronger and more covalent than the Ti-A(A: Ge and In) bond. Similar results for Ti_2AlX (X:B, C and N) materials were obtained and are mentioned in the main text.

The electronic properties of hexagonal Ti_2AX (A: Ge, In and X: B, C or N), including the band structure, the total and projected electronic local density of states, and the Fermi surface are shown figure S1 (for the previously known superconductors – Ti_2GeC, Ti_2InC and Ti_2InN) and figure S3 (for the currently unknown superconductors – Ti_2GeB, Ti_2GeN and Ti_2InB). From these figures, it is obvious that all have metallic characteristics, with the Ti 3d states dominating the density of states at the Fermi energy $N(E_F)$. The calculated $N(E_F)$ values are summarized in table S2 and are in broad agreement with available theoretical results[2, 3, 8, 9].

The Fermi surfaces of Ti_2AX (A: Ge, In and X: B, C or N) compounds are given in figure S1 and figure S3, and qualitatively agree with the previously theoretical results for Ti_2GeC and Ti_2InC[10, 11]. As expected, the Fermi surface of Ti_2InX (X:B, C, and N) compounds are extremely similar to that of Ti_2AlX (X:B, C, and N) compounds described in the main text since Al and In are isoelectronic. Ti_2GeC and Ti_2GeN compounds, like Ti_2AlX (X:B, C, and N) materials, have six Fermi surface sheets, whereas Ti_2GeB has five sheets. Along the Γ-A direction of Ti_2GeX (X:B, C and N), the Fermi surface is completely prismatic and cylindrical and shows electron-like behaviour, whilst hole-like sheets occur at the corners of the Brillouin zone along the H-K and L-M directions. The first sheet of Ti_2GeC is shaped like an hour-glass and is surrounded by a second sheet that is almost cylindrical. The second sheet of Ti_2GeC is cylindrical-like, but with a hexagonal cross-section. There are also electron and hole-like wing sheets along the H-K direction. Ti_2GeB has the simplest Fermi surface topology, while Ti_2GeN has a more complicated

Fermi surface topology, similar to Ti_2GeC.

B. Phonon and electron-phonon interaction of Ti_2AX (A: Ge, In and X: B, C or N) compounds

The calculated phonon dispersion relations, total and partial vibrational density of states (VDOS) and electron–phonon spectral function for Ti_2AX (A:Ge, In and X: B, C or N) are given in figure S2 and figure S4. These are all dynamically stable because they have only positive phonon modes. The phonon spectrum of Ti_2AX (A: Ge, In and X: B, C or N) is divided into two distinct frequency regions: a low-frequency region up to 12 THz, which contains three acoustic and fifteen optical phonon modes, and a high-frequency region between 15 and 21 THz, which has six optical modes. These two regions are divided by a large gap. In the low-frequency region up to 11THz, there is only a small overlap and hybridization of Ti - Al modes for Ti_2AX (A: Ge, In and X: B, C or N). The VDOS is mainly characterized by the motions of C atoms at high frequencies between 15 and 21 THz, as predicted by their light mass and this region includes two peaks separated by a small gap for Ti_2AC(A:Ge or In) and Ti_2AN(A:Ge or In) atoms, but just one peak for Ti_2AB(A:Ge or In) atoms. In the main text, a similar observation was reported with Ti_2AlX (X:B, C and N) compounds. In the low-frequency region, these materials have a very similar phonon structure. Thus, trends in superconductivity in these materials may be more effected by electronic than phonon properties.

To examine the effect of electron-phonon interaction on the superconducting properties of hexagonal Ti_2AX (A: Ge, In, and X: B, C, or N), the frequency dependence of the Eliashberg spectral function ($\alpha^2 F(\omega)$) and the electron-phonon interaction parameter (λ) are shown in figure S2 and figure S4. Figure S2 and figure S4 show that $\lambda(\omega)$ is dominated by the low frequency region. Values for the logarithmic average phonon frequency (ω_{ln}) and λ are shown in table S2. These values are then used with a fixed value of the reduced Coulomb parameter (μ^*=0.13) to calculate the superconducting transition temperature T_c using the Allen-Dynes modification of the McMillan formula. T_c values of Ti_2GeX (X:B, C and N) materials are calculated as 5.0, 9.4 and 11.6 K, respectively. For Ti_2InX (X:B, C and N), T_c is calculated 3.2, 3.7 and 7.0 K respectively. The value of T_c obtained is in excellent agreement with the experimental values where known[7, 12, 13] as can be seen in table S2.

[1] N. A. Phatak, S. K. Saxena, Y. Fei, and J. Hu, Synthesis and structural stability of Ti_2GeC, Journal of alloys and compounds **474**, 174 (2009).

[2] M. Assadi and H. Katayama-Yoshida, Native point defects in Ti_3GeC_2 and Ti_2GeC, Computational Materials Science **128**, 103 (2017).

[3] A. Candan, S. Akbudak, Ş. Uğur, and G. Uğur, Theoretical research on structural, electronic, mechanical, lattice dynamical and thermodynamic properties of layered ternary nitrides Ti_2AN (A= Si, Ge and Sn), Journal of Alloys and Compounds **771**, 664 (2019).

[4] G. Surucu, Investigation of structural, electronic, anisotropic elastic, and lattice dynamical properties of MAX phases borides: An Ab-initio study on hypothetical M_2AB (M= Ti, Zr, Hf; A= Al, Ga, In) compounds, Materials Chemistry and Physics **203**, 106 (2018).

[5] M. Barsoum, J. Golczewski, H. Seifert, and F. Aldinger, Fabrication and electrical and thermal properties of Ti_2InC, Hf_2InC and $(Ti, Hf)_2InC$, Journal of Alloys and Compounds **340**, 173 (2002).

[6] Y. Medkour, A. Bouhemadou, and A. Roumili, Structural and electronic properties of M_2InC (M= Ti, Zr, and Hf), Solid State Communications **148**, 459 (2008).

[7] A. Bortolozo, G. Serrano, A. Serquis, D. Rodrigues Jr, C. Dos Santos, Z. Fisk, and A. Machado, Superconductivity at 7.3 K in Ti_2InN, Solid State Communications **150**, 1364 (2010).

[8] A. Bouhemadou, Calculated structural, electronic and elastic properties of M_2GeC (M= Ti, V, Cr, Zr, Nb, Mo, Hf, Ta and W), Applied Physics A **96**, 959 (2009).

[9] X. He, Y. Bai, Y. Li, C. Zhu, and M. Li, Ab initio calculations for properties of MAX phases Ti_2InC, Zr_2InC, and Hf_2InC, Solid State Communications **149**, 564 (2009).

[10] I. Shein and A. Ivanovskii, First-principles study of electronic band structure and elastic properties of superconducting nanolaminate Ti_2InC, arXiv preprint arXiv:0811.2082 (2008).

[11] M. Hadi, M. Roknuzzaman, F. Parvin, S. Naqib, A. Islam, and M. Aftabuzzaman, New MAX phase superconductor Ti_2GeC: A first-principles study, Journal of Scientific Research **6**, 11 (2014).

[12] A. Bortolozo, O. Sant'Anna, C. Dos Santos, and A. Machado, Superconductivity in the hexagonal-layered nanolaminates Ti_2InC compound, Solid State Communications **144**, 419 (2007).

[13] A. Bortolozo, O. Sant'Anna, C. Dos Santos, and A. Machado, Superconductivity at 9.5 K in the Ti_2GeC compound, Materials Science-Poland **30**, 92 (2012).

[14] H. Fu, W. Liu, Y. Ma, and T. Gao, Prediction study of the axial compressibility, anisotropy and dynamic properties for single crystal Ti_2GeC, Journal of alloys and compounds **506**, 22 (2010).

[15] Y. Zhou, H. Dong, X. Wang, and S. Chen, Electronic structure of the layered ternary carbides Ti_2SnC and Ti_2GeC, Journal of Physics: Condensed Matter **12**, 9617 (2000).

[16] S. Cui, W. Feng, H. Hu, Z. Lv, G. Zhang, and Z. Gong, First-principles studies of the electronic and elastic properties of Ti_2GeC, Solid state communications **151**, 491 (2011).

[17] M. Gamarnik and M. Barsoum, Bond lengths in the ternary compounds Ti_3SiC_2, Ti_3GeC_2 and Ti_3GeC, Journal of materials science **34**, 169 (1999).

[18] A. Ganguly, M. Barsoum, and J. Schuster, The 1300° C isothermal section in the Ti–In–C ternary phase diagram, Journal of the American Ceramic Society **88**, 1290 (2005).

[19] I. Shein and A. Ivanovski, Electronic and elastic properties of the superconducting nanolaminate Ti_2InC, Physics of the Solid State **51**, 1608 (2009).

[20] B. Manoun, O. Leaffer, S. Gupta, E. Hoffman, S. Saxena, J. Spanier, and M. Barsoum, On the compression behavior of Ti_2InC,$(Ti_{0.5}, Zr_{0.5})_2InC$, and M_2SnC (M= Ti, Nb, Hf) to quasi-hydrostatic pressures up to 50 GPa, Solid state communications **149**, 1978 (2009).

[21] B. Liu, J. Wang, J. Zhang, J. Wang, F. Li, and Y. Zhou, Theoretical investigation of A-element atom diffusion in Ti_2AC (A= Sn, Ga, Cd, In, and Pb), Applied Physics Letters **94**, 181906 (2009).

[22] I. R. Shein and A. L. Ivanovskii, Elastic properties of superconducting MAX phases from first-principles calculations, Physica Status Solidi (b) **248**, 228 (2011).

[23] A. Ivanovskii, R. Sabiryanov, A. Skazkin, V. Zhukovskii, and G. Shveikin, Electronic structure and bonding configuration of the H-phases Ti_2MC and Ti_2MN (M= Al, Ga, In), Inorganic materials **36**, 28 (2000).

TABLE S1. Structural properties of Ti_2GeX and Ti_2InX (X: B, C and N) for this work in bold, and their comparison with previous experimental and theoretical results.

Phase	a(Å)	c(Å)	z	d_{Ti-Ti}(Å)	d_{Ti-Al}(Å)	d_{Ti-C}(Å)	B(GPa)	$B^{'}$
**Ti_2GeB**	**3.182**	**13.575**	**0.092**	**3.101**	**2.824**	**2.221**	**131.6**	**4.38**
**Ti_2GeC**	**3.089**	**13.007**	**0.088**	**2.919**	**2.753**	**2.125**	**157.6**	**4.43**
Exp[1]	3.078	12.934					211	
Exp[13]	3.078	12.920						
Exp[14]	3.101	13.159						
GGA[2]	3.100	13.020						
LDA[8]	3.054	12.891	0.090				160	4.36
LDA[15]	3.079	12.930						
LDA[16]	3.047	12.776	0.091				173	3.86
Theory[17]	3.081	12.929	0.086					
**Ti_2GeN**	**3.039**	**12.738**	**0.089**	**2.869**	**2.698**	**2.089**	**167.6**	**4.58**
GGA[3]	3.046	12.907	0.089					
**Ti_2InB**	**3.223**	**15.122**	**0.081**	**3.085**	**3.157**	**2.231**	**105.9**	**4.58**
GGA[4]	3.228	14.534	0.081				103.8	
**Ti_2InC**	**3.144**	**14.189**	**0.078**	**2.867**	**3.039**	**2.127**	**131.2**	**4.67**
Exp[5]	3.134	14.077						
Exp[12]	3.132	14.060						
Exp[18]	3.130	14.084						
GGA[6]	3.138	14.193	0.078				128	4.50
GGA[9]	3.140	14.170	0.079				128	
GGA[19]	3.137	14.184	0.078				123	
GGA[20]	3.133	14.100					148	4.20
GGA[21]	3.135	14.182					128	
GGA[22]	3.148	14.207	0.078				124	
Theory[23]	3.132	14.061						
**Ti_2InN**	**3.095**	**13.996**	**0.079**	**2.841**	**2.987**	**2.101**	**136.3**	**7.02**
Exp[7]	3.074	13.975						
Theory[23]	3.080	14.075						

TABLE S2. Density of states at the Fermi level (N(E_F)), logarithmic frequency ($\omega_{\ln}$), the average electron-phonon coupling parameter (λ) and the superconducting transition temperature (T_c in K) for the hexagonal Ti_2GeX and Ti_2InX (X: B, C and N) for this work in bold, and their comparison with available previous experimental and theoretical results.

Phase	N(E_F)(states/eV)	$\omega_{\ln}$ (K)	λ	T_c (K)
**Ti_2GeB**	**2.227**	**274.398**	**0.621**	**4.997**
**Ti_2GeC**	**3.319**	**302.584**	**0.739**	**9.399**
GGA[2]	3.381			
Exp[13]				9.500
LDA[8]	3.200			
**Ti_2GeN**	**5.144**	**281.657**	**0.831**	**11.642**
GGA[3]	3.369			
**Ti_2InB**	**2.324**	**264.436**	**0.498**	**3.238**
**Ti_2InC**	**2.938**	**337.820**	**0.544**	**3.664**
Exp[12]				3.100
GGA[9]	2.870			
GGA[19]	2.446			
Theory[23]	3.670			
**Ti_2InN**	**5.182**	**321.601**	**0.655**	**7.016**
Exp[7]				7.300
Theory[23]	4.020			

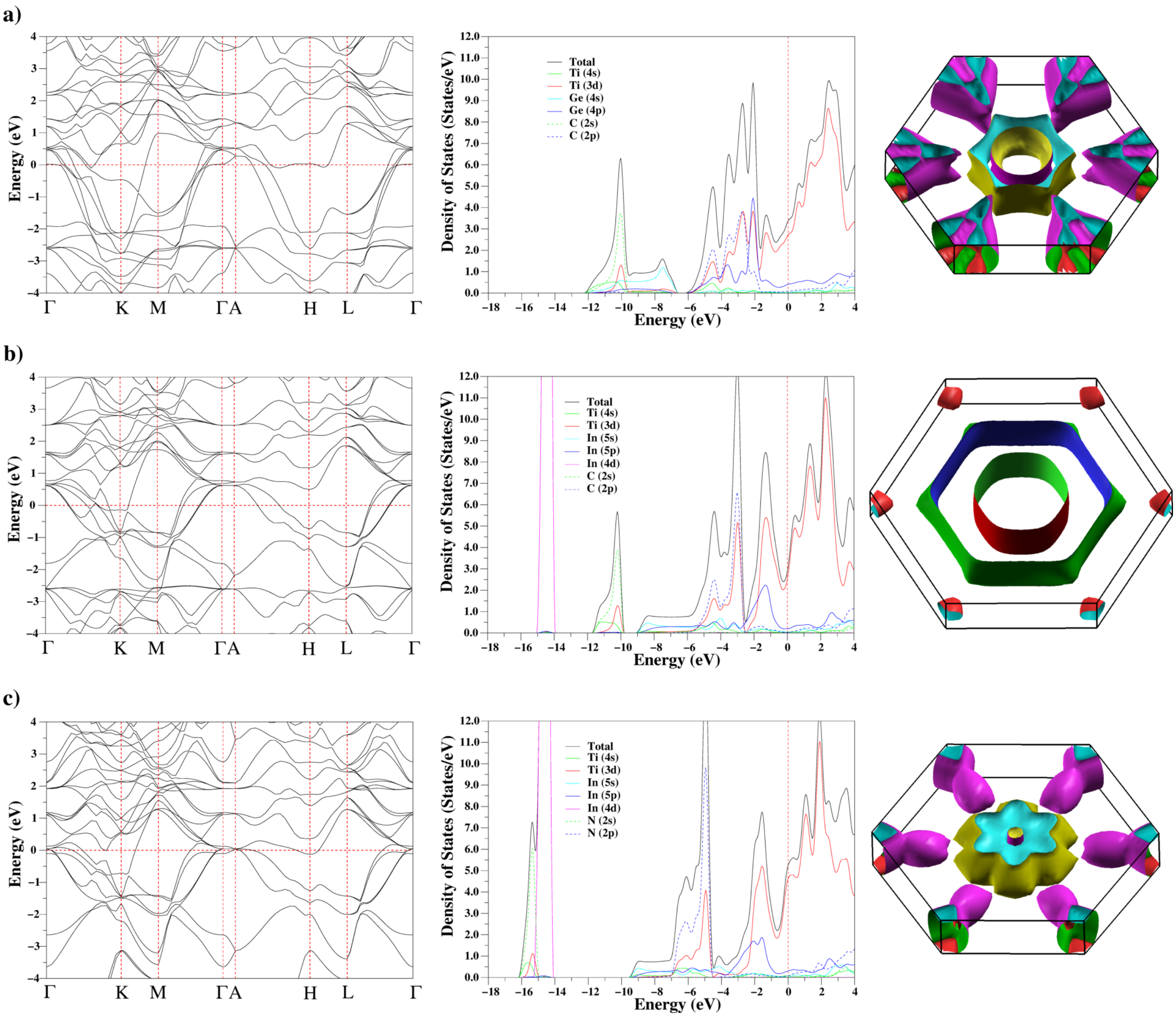

FIG. S1. The electronic band structure, the total and atomic projected electronic local density of states and Fermi surface for the Hexagonal phase of (a) Ti_2GeC (b) Ti_2InC and (c) Ti_2InN.

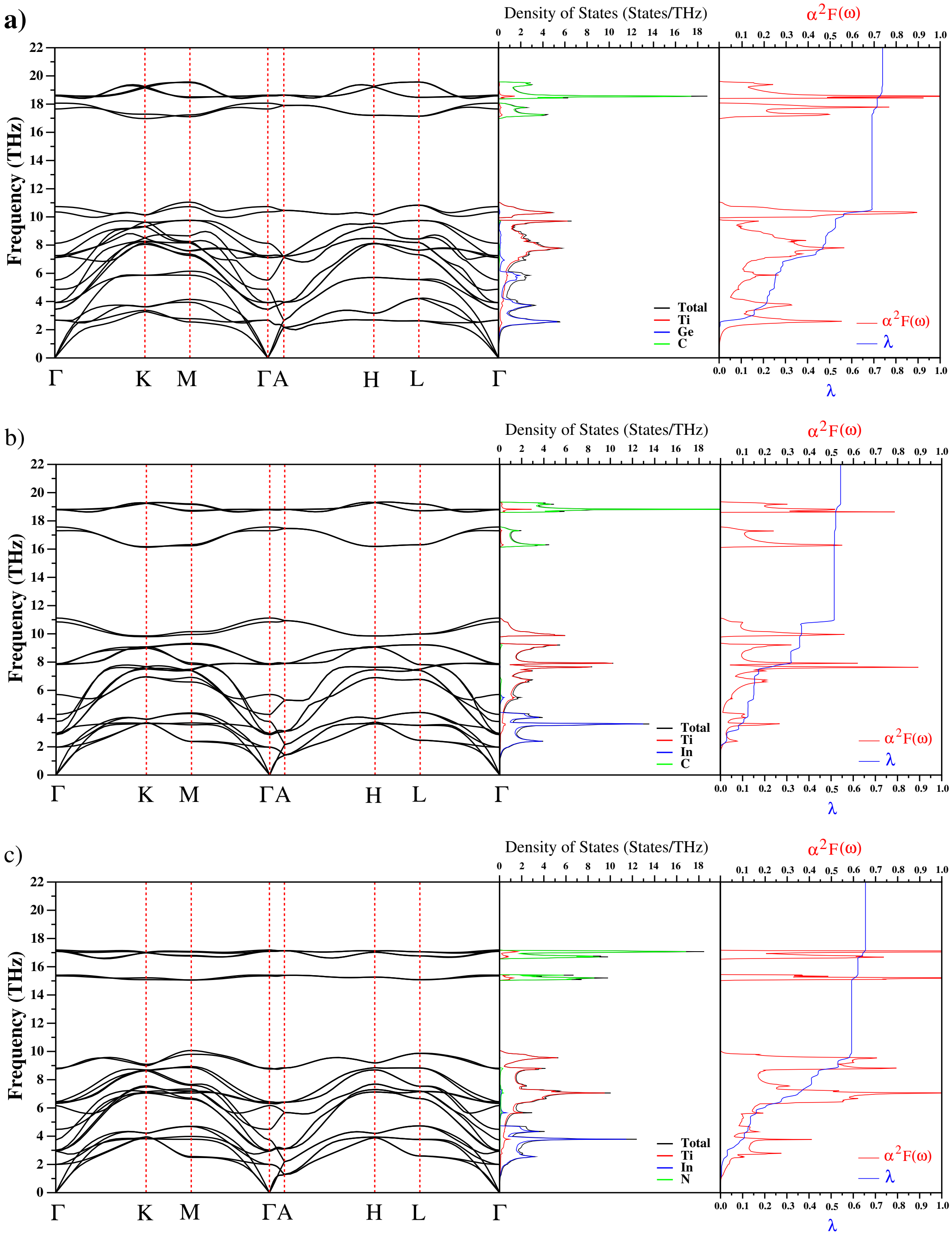

FIG. S2. Phonon dispersion curves, total, partial vibrational density of states and the calculated electron–phonon spectral function $\alpha^2F(\omega)$ (red line) and the variation of the electron–phonon coupling parameter (blue line) with rising frequency $\lambda(\omega)$ of (a) Ti_2GeC (b) Ti_2InC and (c) Ti_2InN

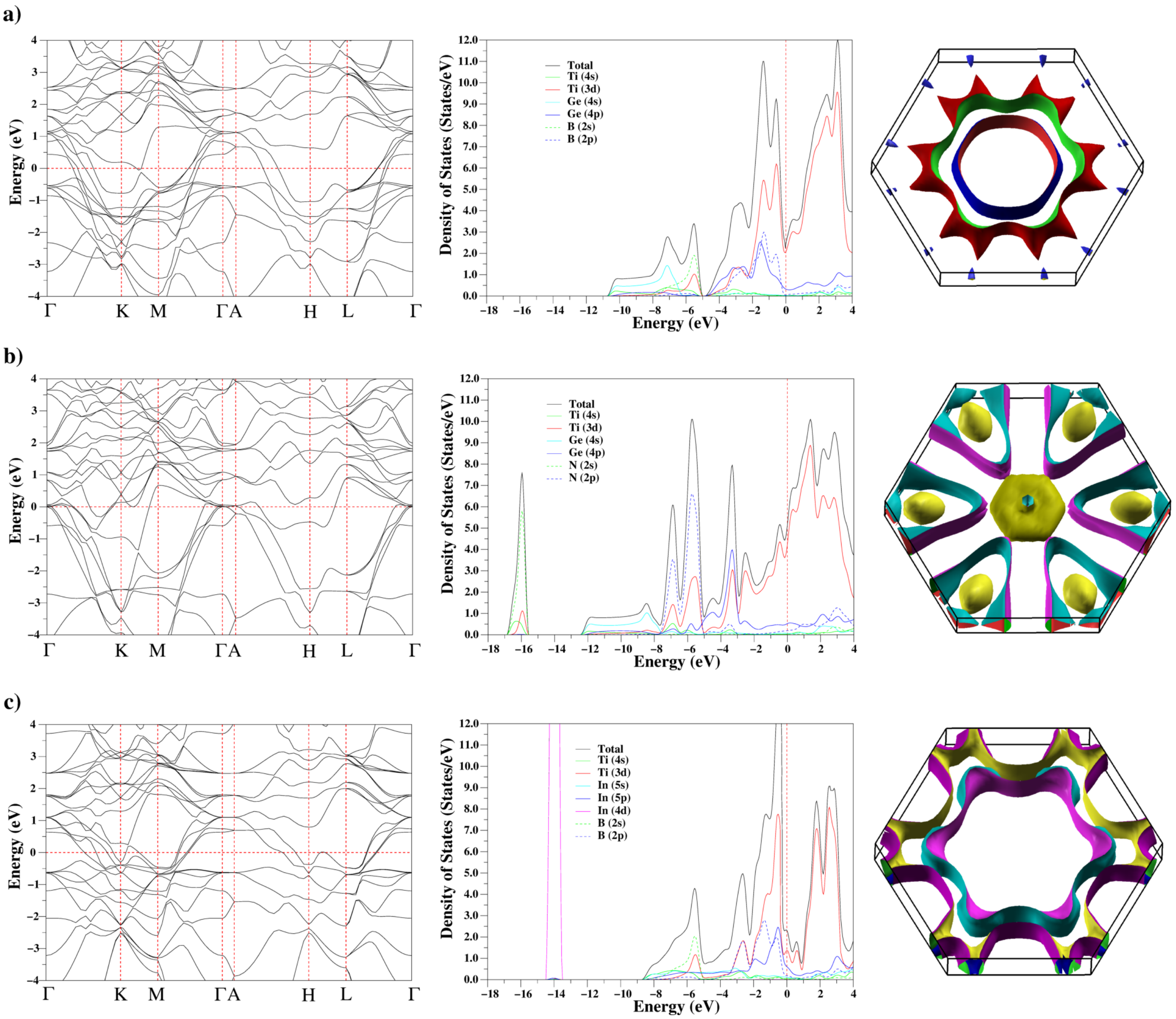

FIG. S3. The electronic band structure, the total and atomic projected electronic local density of states and Fermi surface for the Hexagonal phase of (a) Ti_2GeB (b) Ti_2GeN and (c) Ti_2InB.

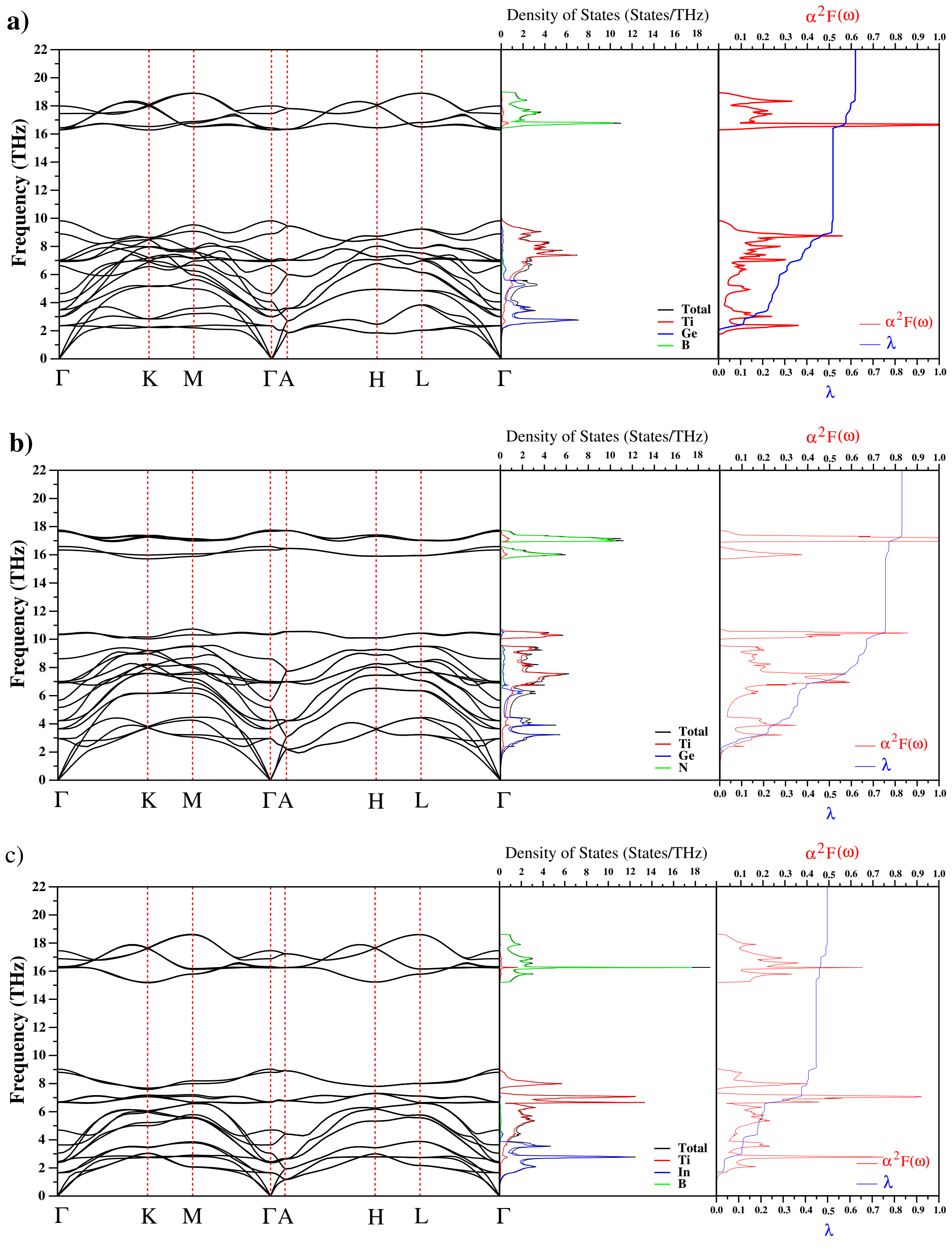

FIG. S4. Phonon dispersion curves, total, partial vibrational density of states and the calculated electron–phonon spectral function $\alpha^2F(\omega)$ (red line) and the variation of the electron–phonon coupling parameter (blue line) with rising frequency $\lambda(\omega)$ of (a) Ti_2GeB (b) Ti_2GeN and (c) Ti_2InB
